# Characterization of unknown genetic modifications using high throughput sequencing and computational subtraction

**DOI:** 10.1186/1472-6750-9-87

**Published:** 2009-10-08

**Authors:** Torstein Tengs, Haibo Zhang, Arne Holst-Jensen, Jon Bohlin, Melinka A Butenko, Anja Bråthen Kristoffersen, Hilde-Gunn Opsahl Sorteberg, Knut G Berdal

**Affiliations:** 1 National Veterinary Institute, Section for Food Bacteriology and GMO, PO Box 750 Sentrum, 0106 Oslo, Norway; 2 School of Life Science and Biotechnology, Shanghai Jiao Tong University, 800 Dongchuan Road, Shanghai 200240, PR China; 3 National Veterinary Institute, Section for Epidemiology, PO Box 750 Sentrum, 0106 Oslo, Norway; 4 University of Oslo, Department of Molecular Biosciences, PO Box 1041, Blindern, 0316 Oslo, Norway; 5 Agricultural University of Norway, Department of Plant and Environmental Sciences, PO Box 5003, 1432 Ås, Norway

## Abstract

**Background:**

When generating a genetically modified organism (GMO), the primary goal is to give a target organism one or several novel traits by using biotechnology techniques. A GMO will differ from its parental strain in that its pool of transcripts will be altered. Currently, there are no methods that are reliably able to determine if an organism has been genetically altered if the nature of the modification is unknown.

**Results:**

We show that the concept of computational subtraction can be used to identify transgenic cDNA sequences from genetically modified plants. Our datasets include 454-type sequences from a transgenic line of *Arabidopsis thaliana *and published EST datasets from commercially relevant species (rice and papaya).

**Conclusion:**

We believe that computational subtraction represents a powerful new strategy for determining if an organism has been genetically modified as well as to define the nature of the modification. Fewer assumptions have to be made compared to methods currently in use and this is an advantage particularly when working with unknown GMOs.

## Background

Genetically modified organisms have been engineered through the stable integration of a recombinant genetic cassette into the genome of a recipient organism. The purpose of generating a genetically modified organism (GMOs) is, like breeding in general, to provide the new variety with novel features, and for introduced traits to be inheritable, the nuclear or organellar genome has to be altered. Protein coding mRNAs represent a causal starting point for most metabolic processes and structural components of a cell, and a cell's pattern of RNA transcription reflects the coding potential of its genome. For a genetic modification to have an effect, it is thus also vital that it changes the coding capacity of the recipient cell.

The strategy most commonly used when generating genetically modified plants that are commercially relevant is to introduce a genetic construct that either confers some kind of advantage when it comes to farming/storage or increases the nutritional quality of the end product. Among the most widely used genetic features are genes that encode herbicide tolerance, insect resistance or improve content of key nutrients . In addition to these trait genes, various selection markers are also usually introduced in order to simplify the process of GMO generation. These genes include herbicide resistance genes such as the bialaphos resistance gene (*bar*) from *Streptomyces hygroscopicus *[1], antibiotic resistance genes such as the neomycin phosphotransferase II gene (*nptII*) from *Escherichia coli *found in the Flavr Savr tomato [2] or positive selection markers such as the phosphomannose isomerase gene (*pmi*) from *E. coli *(used in for instance Golden Rice, see [3]). Careful examination of the pool of transcripts found in a plant should therefore reveal whether or not a plant has been genetically modified.

Recently, a new strategy for identification of foreign nucleic acids (DNA or RNA) called computational subtraction has been described for pathogen discovery in human diseases of unknown etiology [4]. In short, the approach takes advantage of the fact that for a growing number of species the complete genomic sequence has now been generated, and sequencing costs have been dropping dramatically in recent years. Using sequence similarity search algorithms it is thus possible to analyze DNA or RNA sequence data from a sample, compare the sequences against a set of reference sequences, and filter away all the endogenous ('expected') reads, leaving a small collection of sequences that do not appear to stem from the organism in question. This principle appears to work well even when subtracting short sequence tags [5], and should be an efficient way to identify for instance unexpected transcripts.

We have attempted to use high massively parallel pyrosequencing and the concept of computational subtraction to look for allochthonous transcripts in a transgenic line of *Arabidopsis thaliana*. We also explore the concept of computational subtraction *in silico *using expressed sequence tag (EST) data from transgenic rice and papaya.

## Results

The cDNA sequencing of transgenic *A. thaliana *gave a total of 79,990 reads, yielding 17,457,856 bases (average read length: 218 bases) and the raw data were deposited in GenBank's Short Read Archive (SRA) as submission SRA009344: . Sequence tag extraction gave a total of 58,933 high quality 75-basepair sequences. Computational subtraction was performed on the tag datasets and very few *A. thaliana *sequences remained after the second round of subtraction (Table 1). The remaining pool of sequence tags consisted almost exclusively of sequences with a high degree of sequence similarity to the pBI121 vector sequence (Table 1). Thirteen tags did not match the pBI121 vector or our reference transcriptome/genome sequences, but these sequences were all close matches to *A. thaliana *accessions or other plant sequences in the NCBI nt database. The maximum bitscore possible using our megablast settings and sequence length (75 basepairs) was 149, and average score obtained for the remaining 146 sequences was 145.5 when megablast was used against the T DNA (transfer DNA) region of pBI121. For the collection of 75-basepair prokaryotic tags on the other hand, only a very small number of tags were subtracted (Table 1).

**Table 1:** Computational subtraction of 75-basepair sequence tags against *A. thaliana *transcriptome and genome

	**Starting pool of tags**	**Transcriptome megablast**	**Genome megablast**
**Sequenced tags**	58,933 (100%)	5,727 (9.72%)	159 (0.27%)
**pBI121 T DNA tags**	147 (0.25%*)	146 (2.55%*)	146 (91.82%*)
**Prokaryotic tags**	1,000 (100%)	995 (99.5%)	995 (99.5%)

* - percent of total remaining tags that match pBI121 T DNA

**Figure 1:**
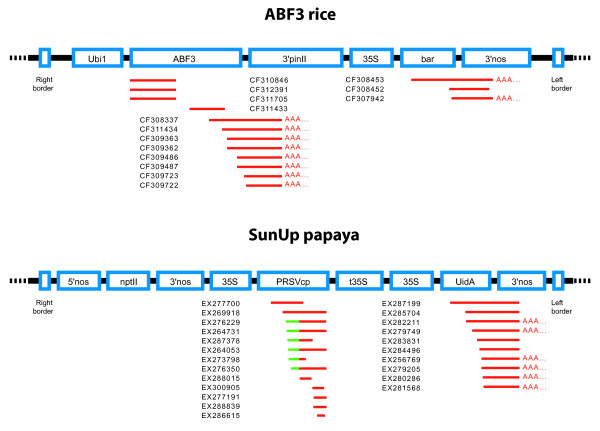
**Construct-derived sequences found in the transgenic EST libraries generated using the ABF3 rice line and SunUp papaya**. 15 sequences were found in the rice library, whereas the SunUp papaya cDNA collection contained 23 construct-derived sequences. Two versions of the papaya ringspot virus coat protein (PRSVcp) transcripts were found, and labeled in green are sequences from the cucumber mosaic virus coat protein (CMVcp) gene. When present in the sequences, poly(A) tails have been indicated and the sequences have been labeled with their GenBank EST accession numbers. Construct maps were modified from [18,19,21] and . Ubi1 - maize ubiquitin promoter 1. ABF3 - abscisic acid responsive elements-binding factor 3. 3'pinII - 3' region of potato proteinase inhibitor II. 35S - Cauliflower Mosaic Virus (CaMV) P35S promoter. bar - phosphinothricin acetyltransferase. 3'nos - 3' region of nopaline synthase. 5'nos - 5' region of nopaline synthase. nptII - neomycin phosphotransferase II. PRSVcp - Papaya Ringspot Virus coat protein. t35S - CaMV P35S terminator. UidA - beta glucuronidase.

A number of transgenic EST reads could be identified in both the rice and the papaya sequence collections (Figure 1). Both the trait genes and selection markers seemed to have reasonable expression levels, and some reads from papaya also showed some diversity in the 5' end of the coat protein transcript (Figure 1). The two different sequences found corresponded to two different versions of transgenic papaya; one with the complete transcript from the papaya ringspot virus and one earlier version where a composite sequence comprising a part of the papaya ringspot virus genome as well as a part of the cucumber mosaic virus genome was used [6].

## Discussion

Most of the methods currently used for characterization of (unknown) genetic modifications rely on PCR [7]. This approach assumes some knowledge about the target sequence, as it relies on primer design. High density array-based methods that make fewer assumptions about the nucleic acids to be detected have been suggested and developed [8,9], but even here some basic assumptions have to be made. By using high throughput sequencing of either a cDNA or a genomic/organellar DNA library, it should be possible to detect any novel transcript or genetic construct. The exception would be if one works with cDNA and the target organisms' only novel feature on the expression level is the increased or reduced expression of an otherwise endogenous gene [10].

Computational subtraction might also be performed using genomic DNA instead of mRNA. The number of sequences that need to be derived for computational subtraction to be effective when working with transcripts will depend upon the frequency and length of the transgenic mRNA versus the pool of endogenous mRNA and small transgenic transcripts and/or a low level of expression will require deeper sequencing. The same principle applies to computational subtraction using genomic DNA, but here the size of the inserted construct relative to the target genome will be the most important factor [11]. Using *A. thaliana *transformed with pBI121 as an example, the insert size is 6,192 bases (GenBank accession number AF485783) and the genome size of *A. thaliana *is 125,000,000 basepairs [12] (excluding mitochondrial and chloroplast genome). If we had sequenced 58,933 genomic tags, we could have expected only to find <3 sequence tags that had sequence overlap with the insert.

One way to increase the likelihood of picking up GM-specific nucleic acids would be to do an *in vitro *physical subtraction of the DNA/RNA before library preparation. This would reduce the amount of nucleic acids that the sample would have in common with a (wildtype) reference and increase the relative amount of the GM-associated DNA or transcripts. There are kits available for performing suppressive subtractive hybridization based on published techniques [2] and subtractions can also be performed commercially (offered by for instance by Eurofins MWG/Operon, see Products & Services at ).

Regardless of what the starting material for library preparation is, the target organisms' transcriptome and/or genome must be well characterized. Sequence filtering might be done using data from a close relative (see for instance the use of mouse data in [4]), but this alone will not be sufficient when working with a high number of sequence reads. At time of writing, ten large plant genome sequencing projects had been completed: *Arabidopsis thaliana *(thale cress), *Glycine max *(soybean), *Phoenix dactylifera *(date palm tree), *Medicago truncatula *(barrel medic), *Oryza sativa *(rice), *Populus trichocarpa *(black cottonwood), *Sorghum bicolor *(sorghum), *Vitis vinifera *(wine grape), *Carica papaya *(papaya) and *Zea mays *(corn). Many more species are slated to be sequenced in the near future , so we believe that for the major crop species this will not be a limiting factor for long.

A possible example of the potential benefits of such an approach was observed in our collection of downloaded EST libraries where a library from a unpublished project entitled 'Subtractive cloning of differentially expressed mRNA from transgenic rice plants' was found (library name: Oryza sativa cv. Pusa Basmati-1). This library comprised only 9 sequences, but even with this small number a reads, a transgenic EST could be detected. The 242 basepair sequence found (accession number AJ309294) was a 100% match with the gene trapping Ds/T-DNA vector pDsG8 designed for insertion mutagenesis in rice [13].

The data generated in this study can also be used to search for other novel transcripts than those that represent transgenic candidates. Careful examination of the 5.568 transcripts that were found that did not match the reference *A. thaliana *transcriptome but matched the genome sequence well (Table 1; 5,727-159 = 5,568), revealed several potentially novel, spliced endogenous genes (data not shown). We do not believe that these transcripts are directly linked to the genetic modification, but this merely demonstrates how versatile data generated using high throughput sequencing of cDNA libraries can be.

## Conclusion

As the amount of available sequences data increases and DNA sequencing costs drop, we believe that a sequencing-based approach using computational subtraction will be feasible for the detection, characterization and risk assessment of genetic modifications. In this pilot study we have shown that transgenic cDNA can be detected using genetically modified plants as a model system.

## Methods

### Plant growth and RNA isolation

*A. thaliana *seeds from plants vacuum infiltrated with *Agrobacterium *[14] carrying the pBI121 *35S:GUS *Ti plasmid (also includes the *nptII *selection marker; Clontech, Mountain View, CA, USA) were surface sterilized and grown on Murashige and Skoog medium [15] without kanamycin for 10 days in growth chambers at 22°C for 8 h of dark and 16 h of light (100 μEm^-2^s^-1^). 10 day old frozen *A. thaliana *seedlings were grinded using a pestle and mortar in the presence of liquid nitrogen and total RNA was isolated using the Spectrum Plant Total RNA kit (Sigma, St. Louis, MO, USA) following the manufacturer's recommendations. RNA was eluted once in 50 μl of elution solution. Quantification of RNA was done using a NanoDrop ND-1000 Spectrophotometer (Thermo Scientific NanoDrop Products, Wilmington, DE, USA).

### Library construction, sequencing and computational subtraction

The mRNA was DNase I treated using a deoxyribonuclease I kit (Sigma) and subsequently reverse transcribed using the SMART PCR cDNA Synthesis Kit (Clontech). Briefly, first-strand synthesis was done using the 3' SMART CDS Primer II A oligonucleotide and PrimeScript Reverse Transcriptase (Takara Bio Inc., Shiga, Japan) in combination with the SMART II A oligonucleotide. cDNA was amplified using the 5' PCR primer II A, and six 50 μl reactions (150 ng DNase-treated RNA per sample as template) were done using 21 PCR cycles. Amplification products were pooled, phenol/chloroform/isoamyl alcohol extracted and ammonium acetate/ethanol precipitated. The pellet was dissolved in molecular grade water and DNA quantification confirmed that the yield was as expected using this kit and protocol.

Nebulization was used to fragment 5 ug of cDNA. Adaptors where appended to the fragments and one GS LR25 sequencing kit (PTP 25 × 75) was used according to manufacturer's recommendations (Roche Applied Science, Indianapolis, IN, USA). Sequencing and library construction was done at the Centre for Ecological and Evolutionary Synthesis' Ultra-high Throughput Sequencing Platform (University of Oslo, Norway) using the 454 Genome Sequencer FLX System (Roche Applied Science).

From the raw data, tags with high sequence quality were extracted. A 75 basepair window was slid through the reads, and when a window that did not overlap with the SMART PCR cDNA linkers or 454 sequencing key and that had an average sequence quality score [16] above 30 was found, a tag was extracted and the algorithm proceeded to the next read.

Sequence subtractions were performed using megablast [17] against a collection of reference mRNA sequences from *A. thaliana *(TAIR8_cdna, downloaded from The Arabidopsis Information Resource ftp site:  with word size 20, no filter for low complexity regions and a high expect (e) value (1000). All of the sequences that gave a match were removed, and the procedure was repeated using the most recent release of the *A. thaliana *nuclear (downloaded from the National Center for Biotechnology Information ftp site:  as well as the mitochondrial and chloroplast genome (NC_000932 and NC_001284, respectively).

In order to test the robustness of the subtraction, a random collection of 75-basepair sequences was extracted from a set of 200+ completely sequenced bacterial genomes. Trait genes used in biotechnology are often of prokaryotic origin, and we used this to simulate what would happen if expression of an unknown prokaryotic gene was to be detected in a pool of endogenous *A. thaliana *transcripts.

### Rice and papaya EST libraries

To test the feasibility of finding cDNA sequences derived from inserted GMO cassettes in a transcript libraries prepared from other plant species, we searched the National Center for Biotechnology Information (NCBI) EST database  for sequence collections derived from genetically modified plants. Focusing on transgenic lines that had an associated publication and that did not merely overexpress endogenous genes we ranked all the libraries found according to size. The largest library was from genetically modified papaya (*Carica papaya*). This cDNA sequence collection had been compiled as a part of the work to characterize the SunUp papaya genome and transcriptome [18]. The six sets of papaya data contained a total of more than 75,000 sequences (EST libraries PY01-PY06; ). The second largest library found (UniGene library 14238) had been derived from GM rice (*Oryza sativa*) and contained 5,455 sequences. It was an unpublished part of a 2005 article by Dr. Oh and colleagues ([19] and Dr. Yeon-Ki Kim, personal communication). The rice line had been transformed with a construct containing the abscisic acid responsive element binding transcription factor 3 gene (*ABF3*) from *A. thaliana *as well as the *bar *gene (phosphinothricin acetyltransferase) as selection marker.

Unfortunately, neither of these two sequence collections appeared to have been filtered for sequence quality or accurately trimmed to remove cloning vector sequences before being submitted. This made efficient computational subtraction intractable (in spite of both the rice and papaya genomes being publicly available), so we decided to instead specifically screen the two libraries for the presence of non-endogenous transcripts (as opposed to removing endogenous transcripts through filtering). The EST sequences were analyzed using BLAST sequence similarity searches and the information that could be obtained from the published ABF3 rice and SunUp papaya GMO cassette construct maps (see references above), the GMO Detection Method Database [20] and the nt sequence collection hosted by NCBI.

## Authors' contributions

TT conceived the idea and wrote the final version of the manuscript. HZ prepared cDNA libraries, JB and ABK did the computational subtraction, MAB and HGS provided mRNA from transgenic *A. thaliana *varieties and AHJ provided funding, supervised and guided the project together with KGB. All authors read and approved the final manuscript.
